# Collaborative Power of Nrf2 and PPAR*γ* Activators against Metabolic and Drug-Induced Oxidative Injury

**DOI:** 10.1155/2017/1378175

**Published:** 2017-08-27

**Authors:** Choongho Lee

**Affiliations:** College of Pharmacy, Dongguk University, Goyang 10326, Republic of Korea

## Abstract

Mammalian cells have evolved a unique strategy to protect themselves against oxidative damage induced by reactive oxygen species (ROS). Especially, two transcription factors, nuclear factor erythroid 2p45-related factor 2 (Nrf2) and peroxisome proliferator-activated receptor *γ* (PPAR*γ*), have been shown to play key roles in establishing this cellular antioxidative defense system. Recently, several researchers reported ameliorating effects of pharmacological activators for these Nrf2 and PPAR*γ* pathways on the progression of various metabolic disorders and drug-induced organ injuries by oxidative stress. In this review, general features of Nrf2 and PPAR*γ* pathways in the context of oxidative protection will be summarized first. Then, a number of successful applications of natural and synthetic Nrf2 and PPAR*γ* activators to the alleviation of pathological and drug-related oxidative damage will be discussed later.

## 1. Reactive Oxygen Species and Human Diseases

Mammalian cells have evolved to utilize oxygen as a final electron acceptor to support their energy metabolism in the mitochondria. As a consequence, they need to deal with a group of unwanted oxygenated byproducts, which are generated during this oxygen-dependent metabolic process. In some cases, environmental stress such as UV or heat exposure also has been attributed to their production. Due to their detrimental nature, these oxygenated byproducts are collectively referred to as highly reactive oxygen species (ROS). Their typical examples include superoxide (O_2_^−^), hydrogen peroxide (H_2_O_2_), hydroxyl radical (OH^−^), and singlet oxygen [1]. A number of cellular metabolic enzymes, such as nicotinamide adenine dinucleotide phosphate (NADPH) oxidase, xanthine oxidase, and nitric oxide synthase (NOS), have been shown to be directly involved in ROS production [2]. Although a certain level of ROS is thought to be necessary for efficient signaling in various cellular pathways [3, 4], most of ROS are generally considered to be harmful due to their damaging effects on essential building blocks of cellular metabolism. For this reason, mammalian cells have developed multiple defense systems to work against this ROS-mediated oxidative stress. One of these antioxidative defense mechanisms is to create a highly reducing intracellular environment to neutralize ROS reactivity before their attack to cellular macromolecules [5].

A growing body of evidence strongly suggests an etiological role of oxidative stress-associated inflammation and cell death in the development of many human diseases [6–11]. Especially, oxidative damage has been intimately linked with the pathogenesis of several chronic metabolic disorders such diabetes, atherosclerosis, and hypercholesterolemia [12–14]. In addition, insufficient cellular protection against oxidative stress also has been ascribed as another contributing factor for developing various liver, kidney, brain, and skin diseases [15–17]. On top of this, oxidative stress was even demonstrated to play a major role in exhibiting many clinically relevant side effects of various pharmacological agents. Therefore, efficient reduction of oxidative stress through activation of multiple antioxidative defense systems was envisaged as a promising strategy to improve a wide range of ROS-induced pathological conditions. Recently, several research groups have published a series of encouraging data suggesting effectiveness of combined use of pharmacological activators for two critical antioxidative pathways. They involve two nuclear transcription factors, which are nuclear factor erythroid 2p45-related factor 2 (Nrf2) and peroxisome proliferator-activated receptor *γ* (PPAR*γ*). Stimulation of these two antioxidative pathways by various pharmacological agents turned out to be extremely beneficial for alleviating a variety of ROS-induced metabolic disorders and drug-induced injuries. In this review, general characteristics of Nrf2 and PPAR*γ* pathways in the context of oxidative protection will be summarized first. Then, a number of successful applications of combined or separate use of Nrf2 and PPAR*γ* activators for amelioration of pathological and drug-induced oxidative injuries will be discussed later.

## 2. Nrf2 Pathway against Oxidative Stress

**Figure 1:**
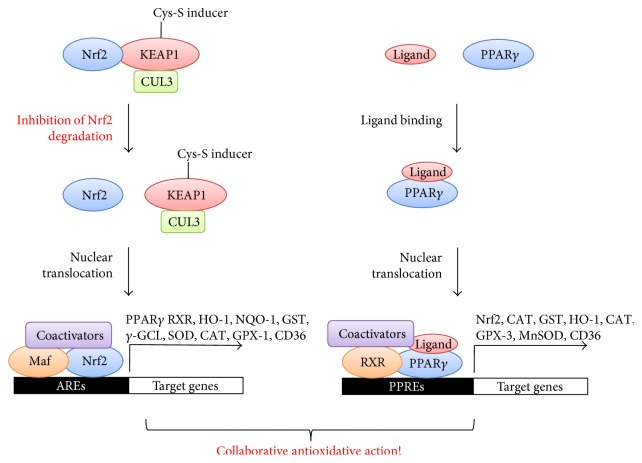
Crosstalk between Nrf2 and PPAR*γ* pathways against oxidative stress. Abbreviations used within the figure are as follows. Nrf2; nuclear factor erythroid 2-related factor 2, KEAP1; Kelch-like ECH-associated protein 1, CUL3; cullin3, PPAR*γ*; peroxisome proliferator-activated receptor *γ*, HO-1; heme oxygenase-1, NQO-1; NAD(P)H quinone oxidoreductase-1, GST; glutathione, *γ*-GCL; *γ*-glutamyl cysteine ligase, SOD; superoxide dismutase, CAT; catalase, GPX; glutathione peroxidase, AREs; anti-oxidant response elements, MnSOD; manganese superoxide dismutase, PPREs; PPAR response element, RXR; retinoid X receptor.

Nrf2 is by far the best characterized transcription factor with an oxidant/electrophile-sensing capability [18]. It is a basic leucine zipper protein with six conserved Nrf2-ECH homology (Neh) domains [5]. Especially, ETGE and DLG motifs located in the second Neh2 domain were shown to play a critical role in its complex formation with another essential component of this pathway, Kelch-like ECH-associated protein 1 (KEAP1) [19]. This Nrf2/KEAP1 complex formation was demonstrated to be necessary for restraining the transcriptional activity of Nrf2 [20]. In regard to its sensing mechanism, KEAP1 acts as a main sensor molecule for oxidative stress in this pathway. It is an adaptor protein for cullin-3-based E3 ubiquitin ligase complex. Redox-sensitive twenty-five cysteine residues of KEAP1 in its linker region function as essential determinants for regulating its ubiquitin ligase activity [21]. Conjugation of a variety of ROS-inducing agents with these cysteine residues leads to inhibition of KEAP1-mediated ubiquitination [22], resulting in stabilization and nuclear translocation of Nrf2. Once transported inside the nucleus, Nrf2 associates with one of small Maf proteins and other coactivators to form a trimetric protein complex. Then, this complex binds to the antioxidant response elements (AREs) in the upstream promoter regions of many cytoprotective and detoxifying genes for their transcriptional activation (Figure 1). Typical examples of Nrf2-regulated genes include *γ*-glutamyl cysteine ligase (*γ*-GCL), NAD(P)H quinone oxidoreductase-1 (NQO-1), glutathione S-transferase (GST), heme oxygenase-1 (HO-1), uridine diphosphate (UDP) glucuronosyl transferase, superoxide dismutase (SOD), catalase (CAT), and glutathione peroxidase-1 (GPX-1) [5, 23–28]. In addition to this KEAP1-dependent mechanism, Nrf2 has been reported to be regulated via a number of KEAP1-independent mechanisms. They include transcriptional activation of Nrf2 gene through aryl hydrocarbon receptor (AHR) and its nuclear translocator (ARNT) binding to xenobiotic response element (XRE) [29], transcriptional activation of Nrf2 target genes through association of NF-*κ*B with ARE, post transcriptional regulation of Nrf2 mRNA with host microRNAs [30, 31], post translational modification of Nrf2 protein by phosphorylation [32, 33], acetylation [34, 35], and ubiquitination [36], and association of Nrf2 protein with novel binding partners [37, 38]. This seemingly complicated transcriptional, epigenetic, and posttranslational control of Nrf2 seems to be designed to fine-tune its antioxidative activity upon redox perturbation in order to minimize damaging effects of oxidative stress on cellular metabolism [39].

## 3. PPAR*γ* Pathway against Oxidative Stress

PPARs are the members of a subfamily of the nuclear receptors and transcription factors. In general, they are involved in the regulation of a wide range of cellular processes such as differentiation, development, metabolism, and even oncogenesis [40–43]. Originally, peroxisome proliferators were found as genotoxic rodent carcinogens due to their proliferative effects on peroxisomes in rats [2, 44, 45]. Interestingly, their peroxisome proliferative activity turned out to be due to their oxidative DNA damage, which was caused by leakage of H_2_O_2_ from peroxisomes [44]. PPAR family genes comprise of three isoforms including PPAR*α*, PPAR*β*/*δ*, and PPAR*γ* [46]. All three subtypes of this PPAR subfamily were found to be highly expressed in mammalian tissues, which were necessary for energy homeostasis [47]. In regard to their signaling mechanisms, once ligand-bound PPARs enter the nucleus, they form a heterodimer with the retinoid X receptor (RXR). Then, they bind to specific PPAR response elements (PPREs) within the promoter region of PPAR-regulated genes [48–50]. Depending on isoforms of PPARs, this PPAR/RXR heterodimer recruits a large protein complex of coactivators to activate the transcription of different sets of PPAR target genes, ultimately leading to a unique physiological outcome (Figure 1) [45].

From the pharmacological point of view, PPAR*γ* has been most extensively characterized as an antidiabetic target [45, 51]. For this reason, it is often called “a glitazone receptor.” In general, PPAR*γ* modulates fatty acid storage and glucose metabolism through stimulation of lipid uptake and adipogenesis by PPAR*γ*-regulated gene expression in fat cells [51]. This was supported by an observation of very limited generation of adipose tissue in PPAR*γ* knockout mice [52]. In addition, PPAR*γ* also has been responsible for pathogenesis of several metabolic and vascular diseases including obesity, diabetes, and atherosclerosis [53–55]. Thanks to their regulatory roles in lipid and carbohydrate metabolism, PPAR*γ* agonists have been widely used in the treatment of hyperlipidemia and hyperglycemia [56, 57]. Although PPAR*γ* was initially regarded as a master regulator of transcription in adipogenesis [58], it was also shown to play additional roles in other biologically relevant processes such as infection and inflammation. In particular, many literatures identified PPAR*γ* as a negative regulator of oxidative stress-induced inflammation under either infectious or pathological conditions [51, 59]. Detailed mechanistic studies also revealed that PPAR*γ* was indeed able to suppress inflammation by transcriptional repression of many well-characterized proinflammatory transcription factors and enzymes such as nuclear factor kappa B (NF-*κ*B), signal transducer and activator of trancription-6 (STAT-6), and activator protein 1 (AP-1), cyclooxygenase-2 (COX-2), and induced nitric oxide synthase (iNOS) [2, 48, 60–62]. Antioxidative function of PPAR*γ* was also reported to be mediated by transcriptional activation of a number of several antioxidant genes such as HO-1, CAT, GPX-3, and manganese superoxide dismutase (MnSOD) through its direct association with PPREs of their promoter regions [48, 49, 63]. For this reason, PPAR*γ* has emerged as a new target for anti-inflammatory and antioxidative pharmacotherapy in many diseases, which are adversely affected by oxidative stress and subsequent inflammation [48, 51, 59].

## 4. Crosstalk between Nrf2 and PPAR*γ* Pathways against Oxidative Stress

Several studies have strongly suggested existence of reciprocal regulation of Nrf2 and PPAR*γ* pathways to reinforce the expression of one another [48, 61, 64]. In this sense, Nrf2 and PPAR*γ* pathways seem to be connected by a positive feedback loop, which maintains the expression of both transcription factors and their target antioxidant genes in a simultaneous manner. Then, what are known about molecular mechanisms for PPAR*γ* regulation by Nrf2? Huang et al. provided insight into this question by identifying PPAR*γ* as a direct target gene induced by Nrf2 transcriptional activation [64]. In line with this finding, several other researchers also reported direct binding of Nrf2 to newly identified AREs in the regions of the PPAR*γ* promoter by using gel shift and coimmunoprecipitation assays (Figure 1) [48, 61, 64, 65]. In their studies, ARE sequences located at −784/−764 and −916 regions of the PPAR*γ* promoter were found to be necessary for Nrf2-regulated PPAR*γ* expression. As supporting evidence to this direct regulation of PPAR*γ* by Nrf2 *in vivo*, PPAR*γ* expression was also found to be markedly lower in Nrf2 knockout mice [64]. Other two studies also reported severely compromised expression of PPAR*γ* in Nrf2 null mice and significantly reduced basal levels of PPAR*γ* by Nrf2 deletion [48, 61]. Then, what is the biological significance of this positive regulation of PPAR*γ* by Nrf2? It was found that Nrf2-regulated PPAR*γ* expression was required for protection against acute lung injury in mice [65]. In this report, PPAR*γ* induction was found to be suppressed in Nrf2-deficient mice in hyperoxia-susceptible manner [65]. This piece of evidence strongly suggests the requirement of positive induction of PAPR*γ* by Nrf2 for the amelioration of acute lung injury induced by hyperoxia. In addition, RXR, another critical component of PPAR*γ* pathway, also turned out to be induced by activation of Nrf2 pathway by using chromatin immunoprecipitation and sequencing experiments [66]. These data further imply the presence of another layer of positive regulation of PPAR*γ* pathway by Nrf2 (Figure 1).

Then, what is known about the opposite pattern of regulation, which is the PPAR*γ* action on Nrf2 pathway? So far, several lines of evidence have raised the possibility of direct involvement of PPAR*γ* in the activation of Nrf2 pathway. Chorley et al. found that PPAR*γ* agonists were able to induce transcription of a set of antioxidative defense genes such as GST, HO-1, and CD36 (Figure 1) [66]. Since these PPAR*γ*-regulated genes belong to a group of Nrf2-regulated genes, this observation strongly suggests direct regulation of Nrf2 pathway by PPAR*γ*. In support of this hypothesis, expression of Nrf2 was also shown to be reduced by knockdown of PPAR*γ* in a mouse model [39]. Kvandova et al. even reported the presence of putative PPREs in the promoter regions of Nrf2 gene [2] (Figure 1). This finding further implies possibility of direct binding of PPAR*γ* on Nrf2 promoter for positive regulation of Nrf2 pathway. On the other hand, collaborative action of both Nrf2 and PPAR*γ* transcription factors on a single target gene also seems to be plausible since GST promoter was found to possess both ARE and PPRE sequences to allow for simultaneous stimulation of its transcription [2]. Therefore, concurrent activation of both Nrf2 and PPAR*γ* pathways by different combinations of pharmacological agonists seems to be possible to achieve the maximum levels of antioxidative state for full protection against the harmful effects of ROS (Figure 1).

## 5. Pharmacological Targeting of Nrf2 and PPAR*γ* Pathways

Many endeavors to pharmacologically manipulate Nrf2 and PPAR*γ* pathways have been shown to be successful in different kinds of *in vitro* as well as *in vivo* disease models. In order to take full advantage of the collaborative action of these two critical antioxidant pathways for alleviation of ROS-induced damages in various metabolic diseases and drug-induced injury, many researchers have tried to apply several Nrf2 and PPAR*γ* activators to various disease models. So far, several metabolic diseases including atherosclerosis, diabetes mellitus, and hepatic and renal diseases have been studied in order to test any beneficial effects of these Nrf2 and PPAR*γ* activators on their disease progression. From now on, therapeutic efficacies and toxicities of various Nrf2 and PPAR*γ* activators studied in these metabolic disorders and some drug-induced organ injuries will be summarized first (Table 1). In order to describe Nrf2 and PPAR*γ* activators in a more systematic manner, they were categorized as Nrf2 activator, PPAR*γ* activators, and dual Nrf2 and PPAR*γ* activators based on their target specificities. Additionally, PPAR*γ* activators were further classified as endogenous, synthetic, and natural PPAR*γ* activators based on their origins of synthesis.

### 5.1. Nrf2 Activator

#### 5.1.1. Bardoxolone Methyl

Bardoxolone methyl (BARD) is an orally available semisynthetic triterpenoid [67]. Its chemical structure is based on the scaffold of oleanolic acid, a naturally occurring pentacyclic triterpenoid. According to preclinical studies, BARD was shown to activate Nrf2 pathway for its antioxidant effect. It was also reported to inhibit NF-*κ*B pathway for its anti-inflammatory effect [68]. Wu et al. found that BARD was able to ameliorate ischemic acute kidney injury (AKI) through increased expression of Nrf2, PPAR*γ*, and HO-1 in the mouse model [69]. In this study, BARD was able to exert its positive effect on PPAR*γ* pathway by enhancing the amount of PPAR*γ* mRNA and protein [69]. In regard to its mechanism of action, they found that BARD was able to transcriptionally activate HO-1 gene during ischemic AKI via Nrf2-independent manner. This finding suggests that direct upregulation of HO-1 by PPAR*γ* could be the main mechanism of action for the reduction of AKI by BARD. In spite of its impressive antioxidant activity, BARD failed to pass the third phase clinical trial for the treatment of chronic kidney disease due to a higher rate of heart-related adverse events, including heart failure, hospitalizations, and deaths [70].

#### 5.1.2. Curcumin

Curcumin is a bright yellow plant-derived chemical used as a food additive and supplement. It is a well-known natural Nrf2 activator [71]. Olagnier et al. discovered that several Nrf2 activators were able to upregulate one of scavenger receptors, CD36, leading to the stimulation of phagocytosis of *Plasmodium falciparum*, a causative pathogen for malaria, on human monocyte-derived macrophages in inflammatory conditions [72]. In accordance with this finding, curcumin was also able to increase phagocytosis of *Plasmodium falciparum* through upregulation of CD36 surface expression on monocytes/macrophages [73]. In this study, seven putative AREs were identified in the promoter region of CD36 gene, which explained mode of the transcriptional activation of CD36 gene by curcumin. Inhibition of curcumin-induced Nrf2 protein expression by a general antioxidant molecule, N-acetyl cysteine treatment, resulted in the loss of upregulation of CD36 by curcumin. This further suggested direct involvement of ROS in the activation of Nrf2 pathway by curcumin [73]. Interestingly, curcumin was also able to increase the expression of PPAR*γ* at transcriptional and translational level [73]. This implies that simultaneous activation of both Nrf2 and PPAR*γ* pathways by curcumin may play a role in upregulation of CD36, which can lead to increased phagocytosis of *Plasmodium falciparum* by macrophages.

### 5.2. Endogenous PPAR*γ* Activators

#### 5.2.1. 15-Deoxy-∆12, 14-Prostaglandin J2

15-Deoxy-∆12, 14-prostaglandin J2 (15d-PGJ2) is an electrophilic cyclopentene prostaglandin. It was shown to act as an endogenous ligand for PPAR*γ* [74, 75]. Its highly reactive *α*, *β-*unsaturated carbonyl groups were shown to readily interact and make a covalent bonding with cysteine thiol groups in the ligand-binding domain of PPAR*γ* [74]. 15d-PGJ2 was also demonstrated to be able to increase Nrf2 expression via a PPAR*γ*-dependent manner [48]. Interestingly, cysteines of the linker region of KEAP1 were also shown to be engaged in direct binding of 15d-PGJ2 to KEAP1 [74]. In regard to mechanism for its antioxidative activity, 15d-PGJ2 was shown to protect neurons from homocysteic acid-induced oxidative death via Nrf2-dependent and PPAR*γ*-independent mechanisms [75]. In this study, Nrf2 knockdown in astrocytes abrogated 15d-PGJ2's neuroprotective effect. Under this Nrf2 knockdown condition, 15d-PGJ2 was not able to facilitate induction of Nrf2 target genes. In contrast, knockdown of the PPAR*γ* did not alter the neuroprotective activity of 15d-PGJ2 [75]. Among many Nrf2-regulated genes, HO-1 turned out to play the most critical role in mediating the antioxidative effect of 15d-PGJ2 [75]. Gong et al. also reported protective activity of 15d-PGJ2 against oxidative stress in RAW264.7 mouse macrophages. In this study, they showed that attenuation of cell death by 15d-PGJ2 was due to its positive induction of the mouse HO-1 gene [76]. More specifically, they found that 15d-PGJ2-induced stabilization of Nrf2 was able to mediate transcriptional activation of the mouse HO-1 through Nrf2 binding on its enhancer region. However, this induction of mouse HO-1 expression by 15d-PGJ2 again turned out to be independent of PPAR*γ* pathway [76].

#### 5.2.2. Nitroalkene Fatty Acids

Nitroalkene fatty acids (NAs) are naturally occurring electrophilic derivatives of unsaturated fatty acids. NAs are formed via nitric oxide-dependent oxidative reactions [77]. Bates et al. found that NAs were able to form direct adduct with KEAP1, leading to the activation of Nrf2 pathway. In this report, they reported that NAs were able to display differential transactivation activities toward Nrf2 and PPAR*γ* pathways in a dose-dependent manner [78]. Briefly, activation of PPAR*γ* pathway occurred at nanomolar concentrations of NAs in MCF7 breast cancer cells. However, activation of Nrf2 pathway occurred at much higher concentrations of NAs (≥3 *μ*M) [78]. Based on these results, they concluded that direct activation of PPAR*γ* transcription by NAs would dominate over their electrophilic activation of Nrf2 during antioxidant/protective responses [78]. Of note, both phosphatide 3-kinase (PI3K) and protein kinase C (PKC) activations were also shown to be required for transcriptional activation of Nrf2 and PPAR*γ* pathways by NAs in this study [78].

#### 5.2.3. Nitrated Fatty Acids

Endogenous nitrated fatty acids (NFAs) are produced by nonenzymatic reaction of nitric oxide or its inorganic reaction products with naturally present unsaturated fatty acids [79]. NFAs can act as activating ligands for all three PPARs, particularly with the greatest potency as PPAR*γ* agonists [80]. Reddy et al. found that a nitro-oleic acid, one of the most potent NFAs, was able to diminish severity of lipopolysaccharide- (LPS-) induced acute lung injury in mice [80]. In regard to its mechanism of action, they found that its protective effect against LPS-induced inflammation was mediated by increased transcriptional activity of PPAR*γ*. They also showed that this upregulation of PPAR*γ* by a nitro-oleic acid led to subsequent induction of Nrf2 and decreased transcription of the proinflammatory gene, NF-*κ*B [80].

### 5.3. Synthetic PPAR*γ* Activators

Thiazolidinedione (TZDs) drugs are cognate ligands for PPAR*γ*. They are frequently used for the treatment of type 2 diabetes [45, 49]. TZDs drugs are known to facilitate insulin-mediated adipocyte differentiation by counteracting the negative effects of inflammatory cytokines [81]. In general, TZDs drug treatment was shown to decrease ROS production in vascular smooth muscle cells [2]. Effects of three kinds of synthetic PPAR*γ* activators on oxidative stress-induced disease models have been examined so far [50, 82–84]. They include rosiglitazone (RSG), troglitazone (TG) in combination with cyanidin, and arylidene-thiazolidinedione. Here, their activities against oxidative stress and mechanisms of action for these antioxidative activities will be discussed briefly.

#### 5.3.1. Rosiglitazone

RSG is a member of the TDZs family and a ligand for the PPAR*γ*. Wang et al. found that RSG was able to protect hepatocytes from high glucose-induced toxicity via both PPAR*γ*-dependent and PPAR*γ*-independent manners [50]. In this study, they found that RSG increased the expression of Nrf2 and HO-1 in a PPAR*γ*-dependent manner, leading to the elimination of excessive ROS [50]. In addition, they also found that the inhibitory effect of RSG on ROS generation was related with PKC inactivation. In line with this positive role of RSG in reduction of oxidative stress, Liu et al. also reported that RSG was able to inhibit paraquat- (PQ-) induced acute lung injury in rats [83]. In this study, they found that protection of rats against PQ-induced acute lung injury by RSG was mediated by activating both Nrf2 and PPAR*γ* pathways. They also showed that inhibition of NF-*κ*B activation by RSG was required for the alleviation of PQ-induced acute lung injury [83].

#### 5.3.2. Troglitazone with Cyanidin

Cyanidin is a natural organic pigment found in many red berries. Shih et al. reported that cyanidin in combination with TG was able to prevent H_2_O_2_-induced cytotoxicity in human hepatoblastoma HepG2 and rat normal hepatocyte cells [84]. In this study, they found that antioxidative activities of cyaniding and TG were mediated through activation of mitogen-activated protein kinase (MAPK) and Nrf2 pathways [84]. They also reported that cotreatment of cyanidin and TG was able to transcriptionally upregulate expression of antioxidant and detoxifying genes through activation of ARE-mediated Nrf2 pathway [84]. Based on these results, they suggested simultaneous administration of cyanidin and PPAR*γ* agonists to reverse the metabolic dysfunction-related oxidative damage [84].

#### 5.3.3. Arylidene-Thiazolidinedione

Fair amount of efforts has been devoted to the modification of chemical structures of TZDs in order to reduce their endogenous side effects such as water retention, weight gain, and eyesight problems. Faine et al. found that one of their chemically modified TZDs, the arylidene-thiazolidinedione 5-(4-methanesulfonyl-benzylidene)-3-(4-nitrobenzyl)-thiazolidine-2,4-dione (SF23), possessed a weaker affinity for PPAR*γ* [82]. However, SF23 turned out to have impressive anti-inflammatory and antioxidant properties, which were evidenced by efficient blockage of LPS-induced inflammation and oxidative stress in RAW 267.4 macrophages [82]. SF23 was also able to enhance the mRNA expression of CD36 and suppress the mRNA expression of both iNOS and COX-2. They also reported that SF23 was able to display better antioxidant effects on the LPS-stimulated macrophages than RSG. Interestingly, this antioxidant activity of SF23 was shown to be exerted via an Nrf2-independent manner [82].

### 5.4. Natural PPAR*γ* Activators

#### 5.4.1. Carotenoids

Carotenoids are organic plant pigments with a tetraterpenoid structure. Zhang et al. found that carotenoids were able to inhibit proliferation of K562 cancer cells through induction of cell apoptosis and blockage of cycle progression [85]. Especially, this carotenoid-induced cell cycle arrest was shown to be mediated by increased expression of a cell cycle blocker, p21, and decreased expression of cyclin D1. This antiproliferative effect of carotenoids was shown to be dependent on upregulation of both Nrf2 and PPAR*γ* expression [85]. Based on these results, they concluded that Nrf2 and PPAR*γ* pathways could be activated in order to induce the growth inhibitory effects on cancer cells [85].

#### 5.4.2. Monascin

Monascin is a natural compound obtained from Monascus-fermented products. Beisswenger found that monascin was able to attenuate the hyperglycemic toxicity induced by methylglyoxal (MG). MG is a major precursor of advanced glycation end products, which were well known for their diabetes-inducing activities through impairment of an insulin transcription factor, pancreatic and duodenal homeobox-1 (PDX-1) [86]. The protective activity of monascin against MG-induced diabetes was shown to be mediated through positive modulation of both Nrf2 and PPAR*γ* pathways [87]. In this report, Hsu et al. identified monascin as novel natural Nrf2 and PPAR*γ* agonists by using Nrf2 and PPAR*γ* promoter reporter assays in HepG2 cells. Activation of Nrf2 pathway by monascin also resulted in downregulation of hyperinsulinemia in an oral glucose tolerance test [87]. In their related studies, they also reported that cotreatment of monascin with another Nrf2 activator, allyl isothiocyanate, was able to attenuate MG-Induced PPAR*γ* phosphorylation and degradation through inhibition of the oxidative stress via a PKC-dependent manner [88].

#### 5.4.3. Ankaflavin

Ankaflavin (AK) is a natural pigment isolated from Monascus-fermented products. It was found to possess the PPAR*γ* agonist activity [89]. Lee et al. reported that AK was able to upregulate Nrf2 pathway to attenuate MG-induced diabetes *in vivo* [90]. Although AK failed to alter hepatic Nrf2 mRNA or protein expression, it significantly increased Nrf2 phosphorylation at serine 40. This led to increased transcriptional activation of HO-1 gene. They also found that protective effects of AK against diabetes were mediated by the upregulation of Nrf2 pathway, resulting in induction of glyoxalase and HO-1 [89, 90]. In addition, AK also was able to increase Maf-A and PDX-1 expression through activation of PPAR*γ* pathway. They suggested that this could be one potential mechanism for elevating pancreatic insulin synthesis and improving hyperglycemia by AK in MG-treated rats [89].

### 5.5. Dual Nrf2 and PPAR*γ* Activators

#### 5.5.1. Genistein

Genistein is a primary isoflavone from soybeans [91]. Zhang et al. found that genistein was able to induce activation of both Nrf2 and PPAR*γ* pathways and that this led to attenuation of H_2_O_2_-induced cell injury in transformed human umbilical vein endothelial cells [92]. In this report, dual activation of Nrf2 and PPAR*γ* pathways by genistein was demonstrated by enhanced promoter activity of both Nrf2 and PPAR*γ* reporters by genistein [92]. In regard to its mechanism of action, induction of HO-1 by genistein seemed to mediate its protective effect against oxidative stress [92].

#### 5.5.2. Vitamin E

Vitamin E is a group of compounds including both tocopherols and tocotrienols. Their antioxidant activities have been extensively characterized by many researchers [93]. Bozaykut et al. reported that vitamin E was able to afford protection against hypercholesterolemia-induced atherosclerosis in the rabbit aorta model. In this study, they found that vitamin E was able to show this protective effect through decreased expression of matrix metalloproteinase-1 (MMP-1) and increased expression of PPAR*γ*, GST-*α*, and ATP-binding cassette transporter 1 (ABCA1) in the aortae of cholesterol-fed rabbits [94]. Protein expression of Nrf2 was also increased in both the cholesterol-fed and the vitamin E-supplemented groups. Vitamin E appeared to afford this protection through activation of both Nrf2 and PPAR*γ* pathways, resulting in induction of several antioxidant genes [94].

#### 5.5.3. Olmesartan

Daunorubicin is a chemotherapeutic medication used to treat various kinds of cancer. Oxidative injury has been suspected to play a major role for daunorubicin in inducing chronic nephrotoxicity [95]. Gounder et al. found that olmesartan, an angiotensin II receptor antagonist, which was used for the treatment of high blood pressure, was able to protect against this daunorubicin-induced nephrotoxicity in rats [96]. In this study, they found that olmesartan treatment downregulated phosphorylation of several key signaling molecules such as mitogen-activated protein kinase-activated protein kinase (MAPKAPK), caspase-12, p47, and p67. Olmesartan was also able to upregulate renal expression of PPAR*γ*, B-cell lymphoma-extra large (Bcl-xL), GPX, and Nrf2 [96]. Based on these results, they concluded that positive regulation of both Nrf2 and PPAR*γ* pathways seemed to mediate protective effects of olmesartan against daunorubicin-induced nephrotoxicity.

#### 5.5.4. *α*-Methylene-*γ*-Lactones

Protolichesterinic acid is a lichen paraconic acid with an *α*, *β*-unsaturated lactone moiety. Le Lamer et al. found that protolichesterinic acid derivatives, *α*-methylene-*γ*-lactones, were able to induce expression of Nrf2 target genes such as NQO-1 and HO-1 and PPAR*γ* target genes such as Dectin-1 and CD36 in macrophages. Based on these results, they concluded that *α*-methylene-*γ*-lactones were potent dual activators of both Nrf2 and PPAR*γ* pathways [97]. In regard to more detailed mechanism of action for activation of PPAR*γ* pathway by *α*-methylene-*γ*-lactones, they suggested that *α*-methylene-*γ*-lactones may act as covalent ligands through a Michael addition with a cysteine residue in the PPAR*γ* ligand-binding domain [97].

#### 5.5.5. 18*β*-Glycyrrhetinic Acid

Methotrexate (MTX) is a dihydrofolate reductase inhibitor used for several human malignancies and autoimmune disorders. Due to its prooxidant and nonspecific action, MTX has been reported to induce a variety of adverse effects [98, 99]. 18*β*-Glycyrrhetinic acid (18*β*-GA) is one of the active ingredients of *Glycyrrhiza glabra* (Liquorice). Abd El-Twab et al. reported that 18b-GA supplementation was able to significantly upregulate the mRNA abundance of both Nrf2 and HO-1 in the kidney of MTX-treated rats [100]. 18b-GA administration was also able to downregulate levels of circulating kidney function markers, tumor necrosis factor-*α* (TNF-*α*), kidney lipid peroxidation, and nitric oxide. This protective activity of 18b-GA against MTX-induced kidney injury appeared to depend solely on activation of Nrf2 with no participation of PPAR*γ* pathway [100].

Cyclophosphamide (CP) is a chemotherapeutic agent used to suppress the immune system and cancer. CP-induced ROS generation and oxidative stress have been implicated in its hepatotoxic effects [101]. Mahmoud and Al Dera found that 18*β*-GA acid was able to exert protective effects against CP-induced hepatotoxicity. They also showed that this hepatoprotective activity of 18*β*-GA was mediated through activation of both Nrf2 and PPAR*γ* pathways and suppression of NF-*κ*B pathway [102]. More specifically, 18*β*-GA decreased expression levels of malondialdehyde (MDA), NF-*κ*B, and iNOS and increased expression levels of GSH, GPX, SOD, and CAT [102].

#### 5.5.6. (−)-Epigallocatechin-3-Gallate

(−)-Epigallocatechin-3-gallate (EGCG) is a well-known green tea polyphenolic compound with an antioxidant activity. Ye et al. found that EGCG was able to ameliorate crescentic glomerulonephritis through activation of Nrf2 pathway [103]. In this study, they induced crescentic glomerulonephritis by administration of a rabbit anti-mouse glomerular basement membrane antibody. Under this condition, EGCG-treated mice showed significant reduction in phosphorylation levels of several signaling molecules such as AKT, c-Jun N-terminal kinase (JNK), extracellular signal-regulated kinase (ERK), and p38. EGCG administration also induced a marked increase in the levels of Nrf2, GCL, GPX-1, NQO-1, PPAR*γ*, and silent information regulator 2 (Sir2) protein 1 (SIRT1) in the kidney tissue [103]. All these transcriptional changes induced by activation of both Nrf2 and PPAR*γ* pathways seemed to contribute to amelioration of crescentic glomerulonephritis induced by a glomerular basement membrane antibody.

#### 5.5.7. Mangiferin

Mangiferin is a naturally occurring glucosylxanthone xanthonoid from *Mangifera indica*. Mahmoud-Awny et al. found that mangiferin was able to mitigate gastric ulcer in ischemia/reperfused rats. They also found that mangiferin was able to exert its gastroprotective effect via inducing the expression of Nrf2, HO-1, and PPAR*γ* along with downregulating that of NF-*κ*B [104]. The effect of mangiferin, especially at the high dose, exceeded that was mediated by omeprazole, a proton pump inhibitor [104].

#### 5.5.8. 3-O-Laurylglyceryl Ascorbate

Ascorbic acid is a water-soluble vitamin with an antioxidant activity. A newly synthesized amphipathic derivative of ascorbic acid, 3-O-laurylglyceryl ascorbate, was shown to activate both Nrf2 and PPAR-*γ* pathways [105]. Specifically, 3-O-laurylglyceryl ascorbate was shown to be able to upregulate the expression of mRNAs encoding PPAR-*γ* and Nrf2 and their target genes including *γ*-GCS, HO-1, and NQO-1 [105]. Downregulation of Nrf2 mRNA level in siPPAR*γ*-treated cells further supported the reciprocal positive modulation of Nrf2 and PPAR*γ* pathways. In addition, the effects of 3-O-laurylglyceryl ascorbate on PPAR*γ* and Nrf2 mRNA levels were reduced by PPAR*γ* knock down in normal human epidermal keratinocytes [105]. This suggested that PPAR*γ* played a major role for 3-O-laurylglyceryl ascorbate in inducing transcription of antioxidant genes.

#### 5.5.9. Umbelliferone

Umbelliferone is a natural product of the coumarin family used in sunscreens. Mahmoud et al. reported that umbelliferone was able to confer a protective effect against hepatotoxicity induced by cyclophosphamide (CP), which is an anticancer and immunosuppressive drug [106]. This hepatoprotective activity of umbelliferone was shown to be mediated by upregulation of Nrf2 and PPAR*γ* pathways. In this report, CP-treated rats showed significant downregulation of Nrf2, HO-1, and PPAR*γ.* However, this effect was markedly reversed by umbelliferone treatment [106]. Activation of PPAR*γ* also appeared to inhibit the fibrogenic response to hepatic injury and protect against CP-induced inflammation [106].

#### 5.5.10. *Graptopetalum paraguayense* and Resveratrol

As previously mentioned, advanced glycation end products were generated by nonenzymatic reactions between carbohydrates and proteins and found to cause pancreatic damage and oxidative stress in hyperglycemic patients [107, 108]. Lee et al. used carboxymethyllysine (CML) to induce pancreas dysfunction and hyperglycemia through formation of advanced glycation end products. Using this model, they found that cotreatment of *Graptopetalum paraguayense* (GP) and resveratrol was able to ameliorate CML-induced pancreas damage and hyperglycemia. Especially, resveratrol and ethanol extracts of GP increased insulin synthesis via upregulation of pancreatic PPAR*γ* and PDX-1. Resveratrol and ethanol extracts of GP also strongly activated Nrf2 pathway including GSH and *γ*-GCL to attenuate oxidative stress and improve insulin sensitivity [109].

#### 5.5.11. Cyanidin-3-Glucose and Resveratrol

Cyanidin-3-glucose (C3G) is a natural plant pigment with an anthocyanin structure. Serra et al. found that cotreatment of C3G and resveratrol was able to induce Nrf2 activation leading to increased HO-1 and *γ*-GCL mRNA expression in human colon cancer cells [110]. Resveratrol was also able to increase nuclear levels of PPAR*γ* in cytokine-stimulated cells. Based on these results, they suggested the use of polyphenols as nutraceuticals to lessen intestinal inflammation in patients with inflammatory bowel disease [110].

## 6. Concluding Remarks

In this paper, we have reviewed roles of oxidative stress in the development of human diseases, two major antioxidant signaling cascades such as Nrf2 and PPAR*γ* pathways, their potential crosstalk against oxidative stress, and pharmacological targeting of these two pathways by various Nrf2 and PPAR*γ* activators. Since a growing body of evidence strongly suggests existence of the intimate relationship between oxidative stress and the development of various metabolic disorders and drug-induced organ injuries, discovery of the best combination of Nrf2 and PPAR*γ* activators to achieve the maximal protection against this oxidative stress will be greatly beneficial for alleviating burden of numerous patients suffering from many oxidative stress-induced diseases and side effects of anticancer drugs.

## Figures and Tables

**Table 1:** Summary of Nrf2 and PPAR*γ* pathway activators studied for their potential protective effects on various disease models. Nrf2 and PPAR*γ* pathway activators are categorized based on their target specificities and origin of synthesis. Their compound names, target diseases, effects on experimental models, effect on Nrf2, PPAR*γ*, and other relevant molecules, and their related references are listed accordingly. Abbreviations used within the table are as follows. Nrf2: nuclear factor erythroid 2p45-related factor 2; PPAR*γ*: peroxisome proliferator-activated receptor *γ*; HO-1: heme oxygenase-1; CNS: central nerve system; ARE-luc: antioxidant response element-containing luciferase reporter; PI3K: phosphatide 3-kinase; PKC: protein kinase C; LPS: lipopolysaccharide; NF-*κ*B: nuclear factor kappa B; COX-2: cyclooxygenase-2; MAPK: mitogen-activated protein kinase; iNOS: inducible nitric oxide synthase; GST-*α*: glutathione S-transferase-*α*; ABCA1: ATP-binding cassette transporter 1; MAPKAPK: mitogen-activated protein kinase-activated protein kinase; Bcl-xL: B-cell lymphoma-extra large; NQO-1: NAD(P)H quinone oxidoreductase-1; SOD: superoxide dismutase; GPX: glutathione peroxidase; GST: glutathione; TNF-*α*: tumor necrosis factor-*α*; MDA: malondialdehyde; CAT: catalase; NO: nitric oxide; SIRT1: silent information regulator 2 (Sir2) protein 1; *γ*-GCL: *γ*-glutamyl cysteine ligase.

Category	Compound name	Target disease	Effects on experimental models	Effect on Nrf2	Effect on PPAR*γ*	Effect on others	Reference
Nrf2 activators	Bardoxolone methyl	Kidney disease	Amelioration of ischemic acute kidney injury in mice	Nrf2 ↑	PPAR*γ* ↑	HO-1 ↑	Wu et al. [69]
	Curcumin	Malaria	Increased nonopsonic phagocytosis of *Plasmodium falciparum* in monocytes/macrophages and hepatoma cells	Nrf2 ↑	PPAR*γ* ↑	CD36 ↑	Mimche et al. [73]

Endogenous PPAR*γ* activators	15-Deoxy-D12, 14-prostaglandin J2	CNS disease	Protection against homocysteic acid-induced oxidative death in neurons	ARE-luc ↑	Independent	HO-1 ↑	Haskew-Layton et al. [75]
		Not specified	Attenuation of cell death in RAW264.7 mouse macrophages	Nrf2 ↑	Independent	HO-1 ↑	Gong et al. [76]
	Nitroalkene fatty acids	Not specified	Activation of Nrf2 and PPAR*γ* pathways in human MCF7 breast cancer cells	Nrf2 ↑ (<1 *μ*M)	PPAR*γ* ↑ (>1 *μ*M)	PI3K, PKC ↑	Bates et al. [78]
	Nitrated fatty acids	Respiratory disease	Decreased severity of LPS-induced acute lung injury in mice	Nrf2 ↑	PPAR*γ* ↑	NF-*κ*B ↓	Reddy et al. [80]

Synthetic PPAR*γ* activators	Rosiglitazone	Diabetes	Protection against high glucose-induced toxicity in hepatocytes	Nrf2, ARE ↑	Independent	HO-1 ↑ PKC, COX-2 ↓	Wang et al. [67]
		Respiratory disease	Protection against paraquat-induced acute lung injury in rats	Nrf2 ↑	PPAR*γ* ↑	NF-*κ*B ↓	Liu et al. [83]
	Troglitazone and cyanidin	Liver disease	Protection against H_2_O_2_-induced cytotoxicity in human hepatoblastoma HepG2 and rat normal hepatocytes	Nrf2 and ARE ↑	PPAR*γ* ↑	MAPK ↑	Shih et al. [84]
	Arylidene-thiazolidinedione	Diabetes	Blockage of LPS-induced inflammation and oxidative stress in RAW mouse macrophages	Independent	PPAR*γ* ↑	CD36, HO-1 ↑ iNOS, COX-2 ↓	Faine et al. [82]

Natural PPAR*γ* activators	Carotenoids	Cancer	Inhibition of proliferation of K562 myelogenous leukemia cells	Nrf2 ↑	PPAR*γ* ↑	p21 ↑, cyclin D1 ↓	Zhang et al. [85]
	Monascin	Diabetes	Protection against methylglyoxal-induced toxicity in HepG2 cells and rats	Nrf2 ↑	PPAR*γ* ↑	PKC ↓	Hsu et al. [88]
							Hsu et al. [87]
	Ankaflavin	Diabetes	Protection against methylglyoxal-induced toxicity in HepG2 cells and rats	Nrf2 ↑	PPAR*γ* ↑	Glyoxalase, HO-1 ↑	Lee et al. [90]
							Hsu and Pan [89]

Dual Nrf2 and PPAR*γ* activators	Genistein	Atherosclerosis	Attenuation of H_2_O_2_-induced endothelial cell injury in transformed human umbilical vein endothelial cells	Nrf2 ↑	PPAR*γ* ↑	HO-1 ↑	Zhang et al. [92]
	Vitamin E	Atherosclerosis	Protection against hypercholesterolemia-induced atherosclerosis in rabbit aortae	Nrf2 ↑	PPAR*γ* ↑	GST, ABCA1 ↑	Bozaykut et al. [94]
	Olmesartan	Kidney disease	Protection against oxidative and inhibition of inflammation in daunorubicin-induced nephrotoxicity in rats	Nrf2 ↑	PPAR*γ* ↑	MAPKAPK, caspase-12, p47, p67 ↓ Bcl-xL, GST ↑	Gounder et al. [96]
	*α*-Methylene-*γ*-lactones	Kidney disease	Activation of Nrf2 and PPAR*γ* pathways in mouse peritoneal macrophages from normal and PPAR*γ*-knockout mice against	Nrf2 ↑	PPAR*γ* ↑	Dectin-1, CD36, NQO-1, HO-1 ↑	Le Lamer et al. [97]
	18*β*-Glycyrrhetinic acid	Kidney disease	Protection against methotrexate-induced kidney injury in rats	Nrf2 ↑	Independent	GSH, SOD, GPX, GST, HO-1 ↑, TNF-*α*, MDA, NO ↓	Abd El-Twab et al. [100]
		Liver disease	Protection against cyclophosphamide-induced hepatotoxicity in rats	Nrf2 ↑	PPAR*γ* ↑	MDA, NF-*κ*B, iNOS ↓, GSH, GPX, SOD, CAT ↑	Mahmoud and Al Dera [102]
	(−)-Epigallocatechin-3-gallate	Kidney disease	Protection against crescentic glomerulonephritis induced by administration of rabbit anti-mouse glomerular basement membrane antibody in mice	Nrf2 ↑	PPAR*γ* ↑	SIRT1, *γ*-GCL, GPX1, NQO-1↑, AKT, JNK, ERK, p38 ↓	Ye et al. [103]
	Mangiferin	Gastrointestinal disease	Protection against gastric ulcer in ischemia/reperfused rats	Nrf2 ↑	PPAR*γ* ↑	HO-1 ↑, NF-*κ*B ↓	Mahmoud-Awny et al. [104]
	3-O-Lauryl glyceryl ascorbate	Skin disease	Suppression of oxidative damage induced by H_2_O_2_ and UVB in normal human epidermal keratinocytes	Nrf2 ↑	PPAR*γ* ↑	*γ*-GCS, HO-1, NQO-1, GSH ↑	Katsuyama et al. [105]
	Umbelliferone	Liver disease	Protection against cyclophosphamide-induced hepatotoxicity in rats	Nrf2 ↑	PPAR*γ* ↑	HO-1 ↑	Mahmoud et al. [106]
	*Graptopetalum paraguayense* and resveratrol	Diabetes	Protection against carboxymethyllysine-induced pancreas dysfunction and hyperglycemia in mice	Nrf2 ↑	PPAR*γ* ↑	PDX-1, GSH, GCL ↑	Lee et al. [109]
	Cyanidin-3-glucose and resveratrol	Gastrointestinal disease	Protection against cytokine-stimulated oxidative stress in human colon cancer cells	Nrf2 ↑	PPAR*γ* ↑	HO-1, *γ*-GCS, GSH ↑	Serra et al. [110]
